# Encoding into Visual Working Memory: Event-Related Brain Potentials Reflect Automatic Processing of Seemingly Redundant Information

**DOI:** 10.1155/2013/172614

**Published:** 2013-05-12

**Authors:** Stefan Berti, Urte Roeber

**Affiliations:** ^1^Department for Psychology, Johannes Gutenberg-University Mainz, Wallstraße 3, 55099 Mainz, Germany; ^2^Disciplin of Psychology, School of Health and Human Sciences, Southern Cross University, Hogbin Drive, Coffs Harbour, NSW 2450, Australia; ^3^Institute for Psychology, University of Leipzig, Seeburgstraße 14-20, 04103 Leipzig, Germany; ^4^Biomedical Science, School of Medical Sciences, The University of Sydney, P.O. Box 170, Lidcombe, NSW 1825, Australia

## Abstract

Encoding and maintenance of information in visual working memory in an S1-S2 task with a 1500 ms retention phase were investigated by means of event-related brain potentials (ERPs). Participants were asked to decide whether two visual stimuli were physically identical (identity comparison (IC) task) or belonged to the same set or category of equivalent patterns (category comparison (CC) task). The stimuli differ with regard to two features. (1) Each pattern can belong to a set of either four (ESS 4) or eight (ESS 8) equivalent patterns, mirroring differences in the complexity with regard to the representational structure of each pattern (i.e., equivalence set size (ESS)). (2) The set of patterns differ with regard to the rated complexity. Memory performance obtained the effects of the task instructions (IC versus CC) and the ESS (ESS 4 versus ESS 8) but not of the rated complexity. ERPs in the retention interval reveal that the stimulus-related factors (subjective complexity and ESS) affect the encoding of the stimuli as mirrored by the pronounced P3b amplitude in ESS 8 compared to ESS 4 patterns. Importantly, these effects are independent of task instructions. The pattern of results suggests an automatic processing of the ESS in the encoding phase.

## 1. Introduction

Visual working memory (VWM) performance relies on the ability to maintain relevant information over a short period of time [1] in the service for other mental tasks [2]. This ability is highly restricted by individual differences in capacity [3]. It is assumed that VWM capacity in general is limited to an average of four objects [4, 5]. Bays and colleagues [6] demonstrated that two independent limiting factors may contribute to VWM capacity: encoding limits and storage limits. Hence it is important to decompose processes of memory encoding from processes of memory maintenance in order to investigate VWM processing and performance in more detail. One approach that allows for a separate investigation of encoding and storage function in VWM is the application of event-related brain potentials (ERPs). The human ERP is the averaged EEG activity time-locked to a relevant event (e.g., the presentation of a stimulus), and it composes of sequences of positive and negative components indicating different processing steps. With regard to the present study, the ERP provides two correlates of encoding into and maintenance in working memory: the P3b component reflecting early stages of stimulus processing in the context of memory performance (i.e., encoding, context updating, and categorization; see [7–9]) and slow-wave potentials reflecting maintenance of relevant information over a short period of time (i.e., in a delayed-matching task; see [10–12]).

**Figure 1:**
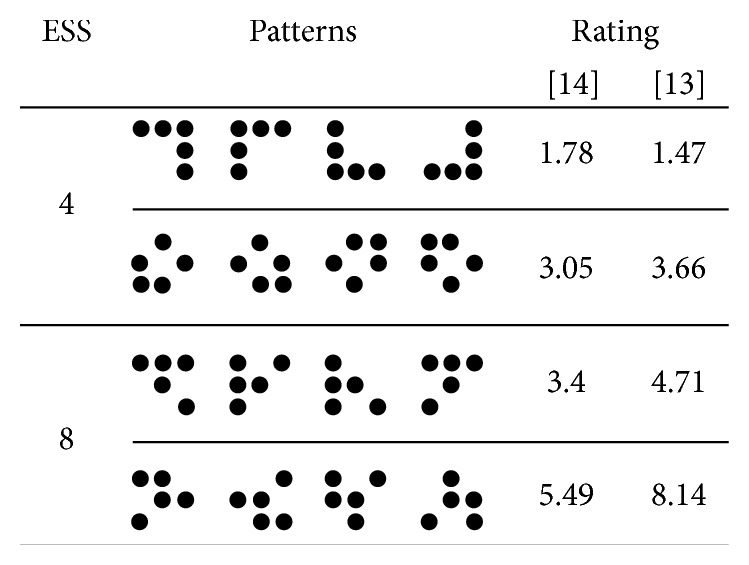
Patterns presented as stimulus material in the S1-S2 memory comparison task. Sixteen five-dot patterns were applied in the present study as S1 and S2. According to the transformation rules (i.e., rotation or mirroring on a diagonal), these patterns are divided into four different sets consisting of four equivalent patterns (*equivalence sets* (ESs)). The different sets of equivalent patterns can consist either of four or of eight patterns and, therefore, have an ES size (ESS) of either four or eight. In the present study, two ESS 4 and two ESS 8 sets were applied; from the ESS 8 sets, four patterns were selected to serve as stimulus material. In addition, with regard to [14, 16], the individual patterns differ in rated complexity (see right column). In the present study, one ES with low and one with high averaged complexity ratings of each set size were applied.

Berti and colleagues [12] demonstrated that encoding and maintenance of information in VWM are differently affected by stimulus properties and task instructions. In detail, participants performed an S1-S2 delayed matching task in which one pattern (S1) had to be compared with another pattern (S2) after a retention phase of 1500 ms. Importantly, there were two conditions. In one condition, S1 and S2 had to be compared irrespective of a potential rotation or mirroring. In other words, S1 and S2 were said to be the same when they were identical or one was the mirror image or resulted from a rotation of the other. In the other condition, the participants' task was to compare S1 and S2 on basis of their physical properties. S1 and S2 were said to be the same only when they were identical. To give an example, the first condition can be partially compared with a comparison based on a category like “letter” (category classification): an upper case “A” and a lower-case “a” then belong to the same category (the letter “a”) and require a “same” response, whereas “A” and “B” do not therefore require a “different” response (see [13]). In the second condition (identity classification), both “A” compared with “a” and “A” compared with “B” require a “different” response. Berti et al. used five-dot patterns to define different stimulus categories (see also [14, 15]). These patterns were constructed on an imaginary 3 × 3 squared grid by leaving no row or column empty [16] yielding a total of 90 patterns (see Figure 1 for some examples). All patterns have the same physical complexity, because each pattern consists of five black dots within the same spatial arrangement. Importantly, the 90 patterns can be divided into 17 sets of patterns that share the same spatial arrangement and can be easily transformed into each other by rotation or reflection on a diagonal. These sets of equivalent patterns (with regard to the rotation and reflection transformation) are referred to as *equivalence sets* (ESs; see [14–16]). In addition, the sets differ in their set sizes comprising 1, 4, or 8 patterns (*equivalence set size* (ESS)). The size of each ES determines the number of possible “same” classifications in the condition with the category classification instruction. This marks a critical feature of this stimulus set: because of the different ESSs, one can manipulate task demands in the category-classification condition while keeping the complexity of the sensory input constant. For the reason that the different ESs are not built on basis of differences in sensory features (because all patterns consist of the same five dots) but on an inherent representational feature patterns (the ES can be represented by one pattern and the two transformational rules), we refer to this feature as the *internal structure* of the patterns.

When it comes to VWM processes, performing the identity classification condition requires the encoding and maintenance of only the presented S1, whereas performing the category classification condition might require the encoding and maintenance of the presented S1 and its ES of all possible transformations: the amount of potential information that can be retrieved from S1 is increased (including the ES or the possible transformations) and varies with the particular ESS of each pattern. If so, condition and/or ESS should influence processing of S1. It is, however, possible that neither task instruction nor ESS affects S1 processing: a possible strategy could be to encode and maintain only S1 and to adapt to different task instructions during retrieval and memory comparison (i.e., using mental rotation in the category classification task [17]). Intriguingly, Berti et al. [12] showed that both ESS (internal structure) and task demands (identity or category classification) affected S1 processing. The most remarkable effect of ESS was obtained at Cz between 500 and 800 ms after S1 onset: ERPs to patterns with an ESS of eight were more positive than ERPs to patterns with an ESS of four reflecting an increased P3b in ESS 8 patterns. Effects of task demands occurred in the slow waves at lateral electrodes (e.g., T8) starting around 500 ms after S1. They were more negative in the identity classification condition than in the category classification condition. This pattern of results suggests that information represented in VWM is not restricted to perceptual properties but can include information related to particular task demands like transformation rules or the preactivation of an ES (see [12, 14, 16]). Moreover, there is evidence that such comprehensive representation of relevant information occurs even when this leads to a seemingly redundant representation: performance in a perceptual classification task (i.e., the to-be-compared objects were presented simultaneously) was affected by the ESS [18]. 

One may assume that more positive P3b in the time window of 500–800 ms for high ESS stimuli in the study by Berti et al. [12] reflects the elaborate encoding of the relevant information into VWM [7–9]. Interestingly, this elaborate encoding occurs irrespective of whether task instructions require this information or not. This interpretation relies on the fact that the stimuli did not differ in physical complexity or other sensory features; therefore, only the internal structure, that is, ESS membership, of these stimuli can account for the differences in the ERPs. On the other hand, the presented visual patterns did differ in their perceived complexity, too, as demonstrated by subjective ratings of the goodness of each individual pattern [14, 16]. Hence the P3b differences might just reflect processing of subjective complexity.

To directly address this issue, we replicated Berti et al.'s study using a different set of five-dot patterns that allowed us control for the factor of subjective stimulus complexity. We asked participants to perform the delayed matching task in two conditions: in the *identity comparison* (IC) condition, the task was to decide whether S1 and S2 were identical (same response) or not (different response); in the *category comparison* (CC) condition, the task was to decide whether S1 and S2 were either identical or could be transformed into each other by rotation or reflection (same response) or not (different response). We used 16 patterns of four different ESs as stimuli (see Figure 1): half of the stimuli originate from sets with an ESS of four patterns (ESS 4) and the other half from sets with an ESS of eight patterns (ESS 8). Within each ESS, we selected one set with low and one set with high complexity ratings (as reported in [14, 16]; for the ratings see Figure 1). Besides the reduction of the number of patterns and ES and the selection of ES patterns with high and with low complexity ratings, there were no further changes compared to the study by Berti et al. [12]. These amendments in the design allow (1) for a replication of the original study and, crucially, (2) for a direct comparison of ESS 4 and ESS 8 patterns of comparable complexity. 

## 2. Materials and Methods

### 2.1. Participants

Twelve students (age range: 19–38 years; mean age 24.8 years; three males; all right handed) of the Chemnitz University of Technology participated voluntarily in the experiment for course credit. According to the Declaration of Helsinki, all gave written informed consent after the nature of the experiment was explained to them. After initial data analysis, three data sets were excluded because of technical problems during recording or of too few artifact-free epochs in the EEG.

### 2.2. Task, Stimuli, and Procedure

Participants were visually presented with two consecutive patterns (see Figure 1) as S1 and S2, separated by a gap (retention phase). After presentation of S2, the participants had to decide whether S1 and S2 were the same (depending on task instructions, see below) or not by pressing one of two buttons as fast and as accurate as possible. In detail, each experimental trial started with the presentation of a fixation cross for 500 ms in the middle of the screen with a medium grey background. After a 300 ms interval with a blank screen, S1 was presented for 150 ms in the middle of the screen, followed by a mask (a black square completely covering S1). After 1500 ms (the retention interval), the mask was replaced by S2, which was again presented for 150 ms. Starting with the presentation of S2, participants had a maximal response window of 2150 ms. The trial ended as soon as a button was pressed or the response window elapsed. The intertrial interval lasted 1000 ms. All stimuli including the fixation cross and the mask were presented in black against a medium grey. S1, S2, and the mask were 3 × 3 cm on the screen (resulting in a visual angle of about 5°).

Figure 1 depicts the 16 five-dot patterns that were used as stimuli. According to the transformation rules (i.e., rotation or mirroring on a diagonal), the patterns were sorted into four different sets of equivalent patterns that can be easily transformed into each other. Each set of equivalent patterns is called *equivalence set* (ES). There were two types of equivalence sets differing in their set size: two of the sets consisted of four equivalent patterns each (ESS 4) whereas the other two sets consisted of eight equivalent patterns each (ESS 8). For the latter, we selected only four patterns per set to present in the experiment; the selection of the patterns was conducted in a way that rotation alone is not sufficient to transform one pattern of an ESS in any other of the patterns of the same ESS. Therefore, one-half of the patterns belong to an ES with an ESS of four and the other half belong to an ES with an ESS of eight (see Figure 1). Critically, within each ESS, patterns differed with regard to their rated complexity [14, 16]: one ESS 4 set and one ESS 8 set contained patterns with low perceived complexity, whereas the other ESS 4 set and the other ESS 8 set contained patterns with high perceived complexity (ratings as reported in [14, 16]). Importantly, the complexity ratings of the high complexity ESS 4 patterns and the low complexity ESS 8 patterns differ only slightly (see Figure 1). This allows us to compare the processing of ESS 4 and ESS 8 patterns that are highly comparable in physical and subjectively perceived complexity.

There were two conditions which differed in their task instructions: in the identity comparison (IC) condition, S1 and S2 were defined as “same” when they were physically identical, whereas they defined as “different” otherwise; in the category comparison (CC) condition, S1 and S2 were defined as “same” when they were identical or one was the mirror image or resulted from a rotation of the other and, therefore, belong to the same set or category of patterns, whereas they were defined as “different” if they did not belong to the same category. This set-up led to a three factorial repeated measurement design with the factors *Task* (IC versus CC), *ESS* (4 versus 8), and rated *Complexity* (low versus high). For each task, participants performed in eight experimental blocks consisting of 48 trials each. In each block, half of the trials required a “same” response and the other half a “different” response. Across all trials, each combination of ESS and complexity was presented with equal probability as S1 and in both conditions 96 trials for each ESS complexity combination. The two conditions were realized in two different sessions on two different days. Half of the participants ran the IC condition in the first session; the other half, the CC condition. Each session started with a short practice block consisting of 24 trials but with a different set of patterns than the ones used in the experimental blocks. Button-to-response mapping was counterbalanced between participants.

### 2.3. Behavioral Data Analysis

For each participant, mean response times (RTs) and mean percentage of correct responses for trials with “same” responses (according to [12]) were calculated separately for both ESS types, both levels of rated complexity, and both tasks; mean RTs were calculated on basis of correct responses only. Behavioral data were evaluated by repeated measure analyses of variance (ANOVA) with the within-subjects factors *Task* (IC versus CC), *ESS* (4 versus 8), and *Complexity* (low versus high).

### 2.4. EEG Recording and Analysis

During the experiment, the EEG was recorded from 17 cap-mounted electrodes (EasyCap FMS, Germany) of the 10–20 system using a Synamps amplifier (Neuroscan, VA). The reference electrode was placed at the left mastoid and the ground electrode was placed at the forehead. In addition, the horizontal and the vertical electro-oculograms (EOGs) were recorded. Both EEG and EOG were recorded continuously in DC mode with a 30 Hz lowpass and a 50 Hz notch filter, with a sampling rate of 250 Hz. The EEG was offline filtered with a 20 Hz lowpass filter, and the ERPs were computed separately for each task-by-ESS-by-complexity combination within a time window from 200 ms before to 1650 ms after S1 onset. The 200 ms prestimulus interval served as a baseline. Epochs with extensive eye-movements (the standard deviation within a 200 ms interval exceeded 40 *μ*V in the EOG and at Fz) were excluded from ERP computation.

Figures 3 and 4 depict the ERPs at three electrode positions that represent the ERP effects best. After visual inspection, the ERPs were divided into three consecutive time windows spanning the S1-S2 retention interval (early P3b: from 340 to 500 ms; late P3b: from 500 to 900 ms; slow potential: from 900 to 1600 ms). Averaged amplitudes within these three time windows were calculated separately for each ESS, task, and complexity level at the electrodes showing the largest effects (early and late P3b: Pz; slow potential: T8) for subsequent statistical analysis. For each time window a *Task* (IC versus CC) × *ESS* (ESS 4 versus ESS 8) × *Complexity* (low versus high complexity) repeated measure ANOVA was calculated. 

## 3. Results and Discussion

**Figure 2:**
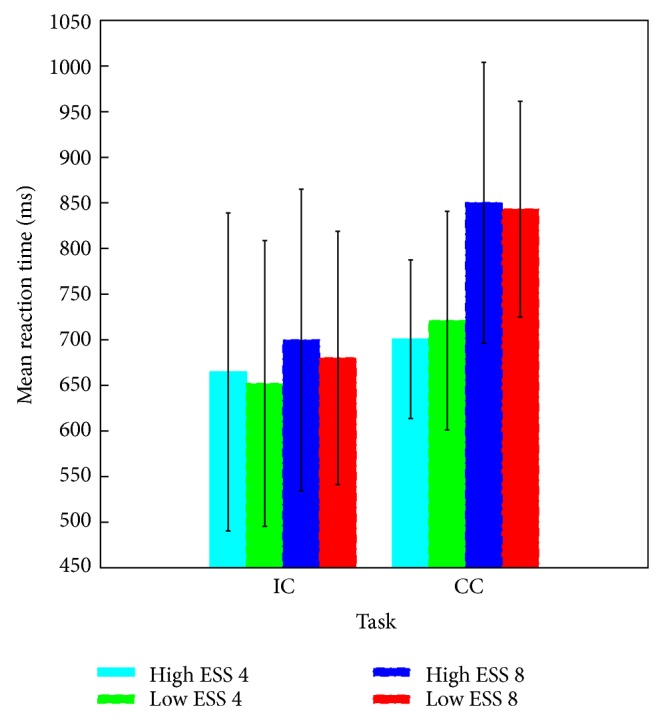
Mean response times (RTs) in the memory comparison task separately for each task-by-ESS-by-complexity combination (error bars represent standard deviation). A task effect and an effect of the ESS are visible with a more pronounced effect of the ESS in the CC compared with the IC task; no effect of the complexity of the patterns is observable. (Note that the RTs were calculated in “same” response trials with correct classifications only.)

 Accuracy in the S1-S2 memory comparison task was high in general (*M* = 99.0%, SD = 1.9), and the percentage of correct responses ranged from 100% to 97.6%. The 2 × 2 × 2 ANOVA revealed significant main effects of ESS (*F*(1,8) = 9.26, *P* < 0.05) and Task (*F*(1,8) = 6.51, *P* < 0.05) reflecting a slight decrease of accuracy for ESS 8 (98.5%, SD = 2.3) compared to ESS 4 (99.6%, SD = 1.1) patterns and in the IC (98.5%, SD = 2.1) compared with the CC (99.6%, SD = 1.4) condition; no other effect was significant. A 2 × 2 × 2 ANOVA computed on the RT data revealed main effects of the factors ESS (*F*(1,8) = 23.70, *P* < 0.01) and *Task* (*F*(1,8) = 8.46, *P* < 0.05), and a significant interaction of these two factors (*F*(1,8) = 14.35, *P* < 0.01) (see Figure 2). No other effect was significant.

Performing memory comparisons with different task demands affected accuracy and RT data. Increased RT when category classifications were required most likely reflects increased task demands in the CC as compared to the IC condition. Importantly, subjective complexity of the patterns did not affect behavioral performance. This pattern of results is in line with earlier findings [14, 15, 18] analyzing and modeling RT data. It remains open whether the factors *Task* and *ESS* affect already the stage of encoding and retention of S1. This question can be addressed by analysis of the ERPs elicited by the S1 presentation.

**Figure 3:**
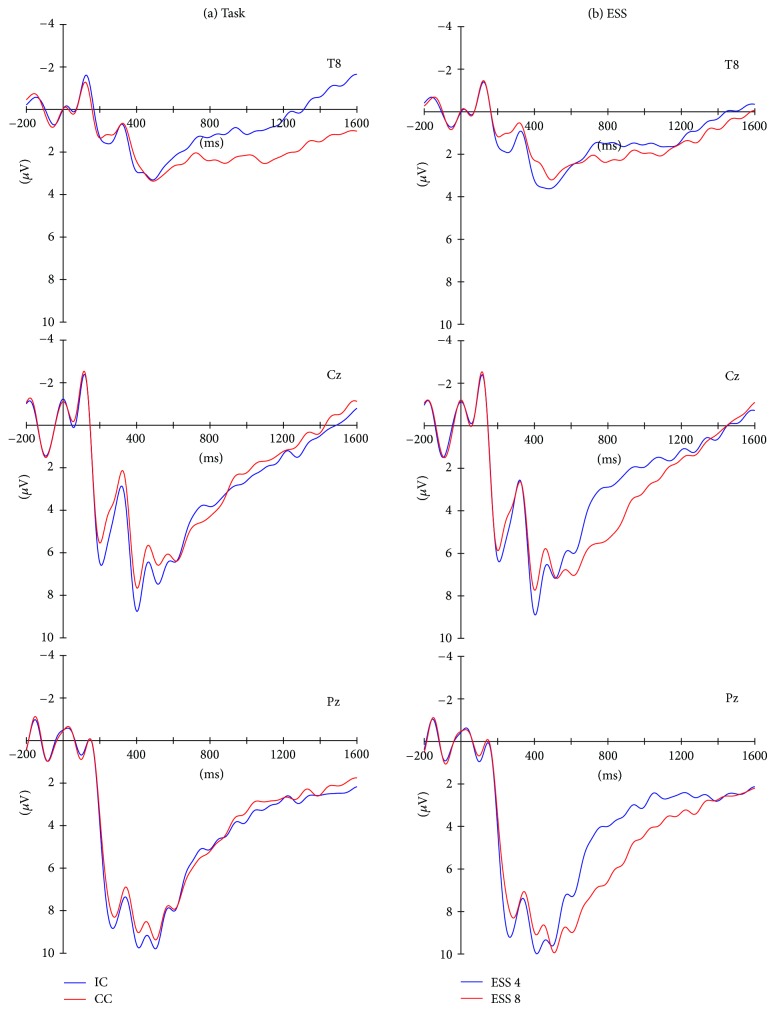
Event-related brain potentials (ERPs) separately for (a) the two different tasks and (b) the two different ESS patterns. (a) A task effect is visible at T8 starting around 600 ms with a more negative slow-wave potential for the CC compared with the IC task. (b) An effect of the ESS is visible at Cz and Pz between 300 and 1200 ms with more positive early P3b for ESS 4 patterns and a pronounced late P3b for ESS 8 patterns.

Figure 3 summarizes the ERPs separately for both tasks (Figure 3(a)) and both ESSs (Figure 3(b)). Visually, the ERP results confirm the findings by Berti et al. [12]: task instruction affects the later phase of the retention interval (slow-wave differences at T8); effects of ESS are confined to the encoding phase (late P3b time window at Pz). However, the *Task* × *ESS* × *Complexity* repeated measure ANOVAs (Table 1) did not reveal an effect of the factor *Task*. *ESS* showed a significant effect only in the late P3b time window (500–900 ms). Interactions of the factors *ESS* and *Complexity* were obtained in the early (340–500 ms) and the late (500–900 ms) P3b time window. No other significant effects were obtained on the 5% level; in particular, the slow wave was not significantly affected by any of the three factors (see Table 1, left column: T8—900–1600 ms).

**Figure 4:**
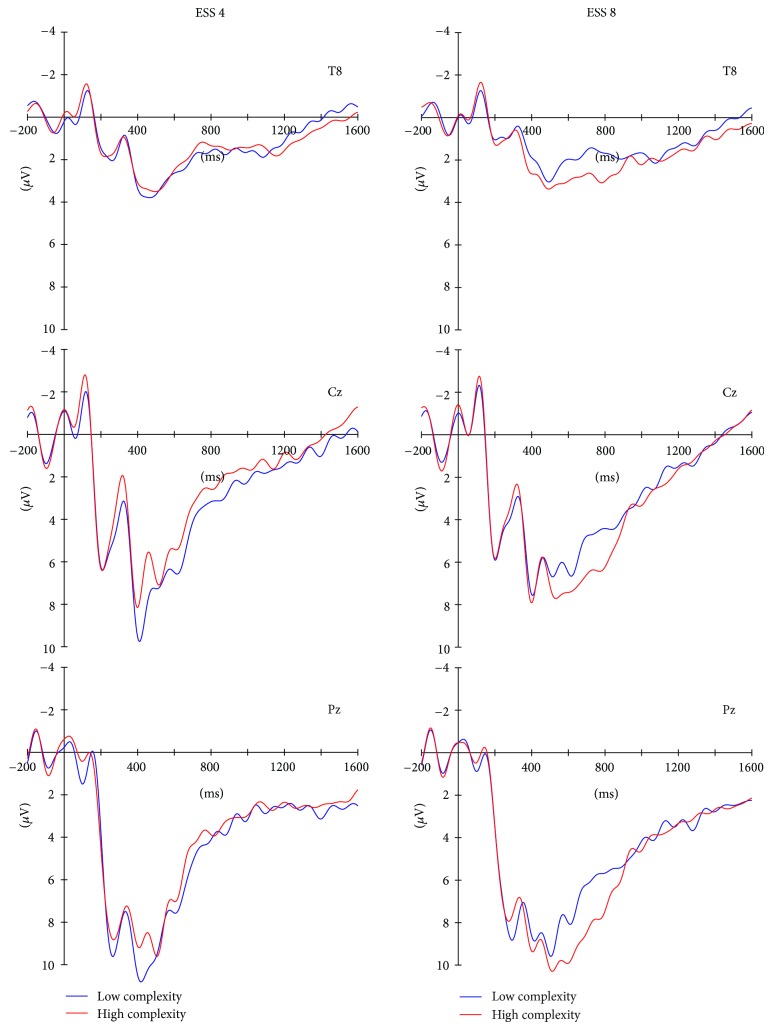
Event-related brain potentials (ERPs) separately for ESS 4 and ESS 8 patterns with low and high complexity. In the early P3b, ESS 4 patterns with low complexity differ from the other three types of patterns (see Cz and Pz left column), showing a pronounced P3b peak. In the late P3b, ESS 8 patterns elicit a pronounced P3b compared with ESS 4 patterns. In addition, the P3b amplitude in ESS 8 patterns is affected by the complexity of the patterns; no effect of complexity is visible in ESS 4 patterns.

Figure 4 depicts the ERPs elicited by S1 separately for the high and low complex ESS 4 and ESS 8 patterns. These ERPs show two remarkable effects. First, ESS4 patterns with low rated complexity elicited a more positive P3b peak around 400 ms at Pz (see also Cz) compared with the other three stimulus types. Presumably, this difference in the P3b peak may reflect that compared to the other three patterns encoding of the low complexity ESS 4 patterns is less demanding and faster and that transition of information in these patterns is already finished at an early stage of encoding [7]. Second, from around 500 ms ERPs to ESS 8 patterns showed enhanced late P3b amplitude than ERPs to ESS 4 patterns. Additionally in the late P3b time window, ERPs to ESS 8 patterns of high rated complexity were larger than ERPs to ESS 8 patterns of low complexity, whereas ERPs to the ESS 4 did not differ depending on rated complexity. Because P3b reflects memory processing on the level of encoding [7–9], the effect of subjective complexity on P3b amplitude is likely to indicate increased encoding demands into VWM for objects with higher external (perceived) and internal (ESS) complexity [12]. This is especially mirrored in the significant *ESS* × *Complexity* interaction in the late P3b and the subsequent differential P3b amplitudes in the late time window for high complexity ESS 8 versus low complexity ESS 8 versus ESS 4 patterns. Finally, a direct comparison of the ESS 4 and ESS 8 patterns with comparable complexity ratings (lower row of ESS 4 and upper row of ESS 8 patterns in Figure 1) in the late P3b time window with a 2 (ESS 4 versus ESS 8) × 2 (IC versus CC) repeated measure ANOVA obtained a significant effect of the factor *ESS*, only (*F*(1,8) = 7.15, *P* < 0.05). This demonstrates that the effect of the internal structure of the patterns as reflected in the different equivalence set sizes affects encoding of information into VWM. In other words, the effect of the ESS in the present study and the study by Berti et al. [12] cannot be attributed to the subjectively perceived complexity of the different patterns. However, in the present study the subjectively perceived complexity of the patterns does affect VWM processing in the encoding phase, too; this effect is confined in ESS 4 patterns to an early encoding phase but is most remarkable during encoding of ESS 8 patterns into VWM.

## 4. Conclusions

 Taken together, the present study demonstrates that encoding of information into VWM is affected by the internal structure of the visual input. The increase of processing demands in the present study is reflected in the late P3b, supporting that P3b in general reflects encoding into WM [7–9, 12]. However, our findings demonstrate that the P3b in the context of VWM processing mirrors not only the encoding of the sensory input but also the activation of additional, relevant information correlated with the sensory input. This conclusion is supported by a recent study by Riby and Orme [19] demonstrating that the semantic context of memory items affects the P3b in the encoding phase. In detail, in a visual short-term memory task, the P3b was increased in patterns which are more easily represented semantically compared to patterns which are not correlated to semantic knowledge [19]. Moreover, in addition to the study of Berti et al. [12], the comparison of ESS 4 and ESS 8 patterns with comparable rated complexity rules out that the effect of the ESS is solely due to differences in the subjectively perceived complexity of the patterns. Importantly, the present results also support the finding that the internal structure of the patterns is processed and encoded automatically [18, 20]: even in the IC task, in which only the physical or sensory input has to be encoded and stored for the subsequent comparison, the ESS affects the P3b. In general, these findings highlight the importance to investigate the encoding processes as an important factor on VWM performance [6]. 

## Figures and Tables

**Table 1:** Summary of the statistical analyses of the ERP results. The 2 × 2 × 2 repeated measure ANOVAs for the three different time windows revealed a significant main effect of the factor ESS in the late P3b time window (500–900 ms at Pz) and significant interactions of ESS and complexity in the early (340–500 ms) and the late (500–900 ms; both at Pz) P3b time windows. No significant effects or interactions of the three factors were obtained for the slow-wave potential.

Time window	Pz—340–500 ms	Pz—500–900 ms	T8—900–1600 ms
Factor	*F*(1, 8)	*F*(1, 8)	*F*(1, 8)

ESS (E)	4.23	10.15∗	<1
Task (T)	1.31	<1	<1
Complexity (C)	<1	3.85	1.23
E × T	1.82	<1	<1
E × C	13.35∗∗	10.91∗	<1
T × C	<1	1.12	2.44
E × T × C	<1	1.06	1.47

Significance levels: ∗*P* < 0.05; ∗∗*P* < 0.01.
